# 

**Published:** 1905-10-07

**Authors:** 


					the hospital. "Cadbury's?the standard of highest purity.''?Lancet. October 7th, 1905.
Cadbury's M ^ Cadbury
WO DRCGS OR ALKALIES "For Purity and Nourishment there
USED. wUwUA 15 nothing superior."?Medical Mag. WUWW
No. 993. Vol, XXXIX. ggpfper!] Saturday,
Oct. 7th", 1905J
[B?b,C?p.,?n T?m.] pR|CE THREEpEHCE,

				

